# 2212. Improving Sexually Transmitted Infection Co-testing in a Large Urgent Care Network

**DOI:** 10.1093/ofid/ofac492.1831

**Published:** 2022-12-15

**Authors:** Allan M Seibert, Michelle M Matheu, Valoree K Stanfield, Matthew Gwiazdon, Naresh Kumar, Kimberly D Brunisholz, Park Willis, Anthony Wallin, Edward A Stenehjem

**Affiliations:** Intermountain Healthcare, Murray, Utah; Intermountain Healthcare, Murray, Utah; Intermountain Healthcare, Murray, Utah; University of Utah, Salt Lake City, Utah; Intermountain Healthcare, Murray, Utah; Intermountain Healthcare, Murray, Utah; Intermountain Healthcare, Murray, Utah; Intermountain Healthcare, Murray, Utah; Intermountain Healthcare, Murray, Utah

## Abstract

**Background:**

Sexually transmitted infections (STIs) remain a serious public health concern. The state of Utah has the lowest percentage of adults 18-64 years-old ever tested for HIV (26.5%) and the lowest percentage tested for HIV in the previous 12 months (6.5%). Increasing HIV testing in Utah is of the utmost importance. Delayed diagnoses and missed testing opportunities for HIV and other STIs exist. Encounters for evaluation of possible gonorrhea (GC) or chlamydia (CT) infection is a critical opportunity to co-test for HIV and syphilis. With continued growth, urgent care (UC) sites are well-positioned to increase STI diagnosis and treatment. We aimed to develop a multi-faceted quality improvement (QI) bundle to increase STI testing in our UC centers.

**Methods:**

Intermountain Healthcare (IH) is a vertically integrated healthcare network predominantly in Utah and operates a network of 35 UC clinics across the state. In 2020, qualitative interviews to evaluate barriers to STI testing were performed with UC clinicians. Based on these interviews a QI initiative was designed and implemented throughout 2021. The bundle included clinician education, electronic health record (EHR) improvements, and automatic referral for patients with a new diagnosis of HIV to an Infectious Diseases (ID) physician (Methods Image 1). We compared co-testing rates before (July 2018 – December 2020) and after the intervention began (March 2021 – April 2022).

**Figure:**
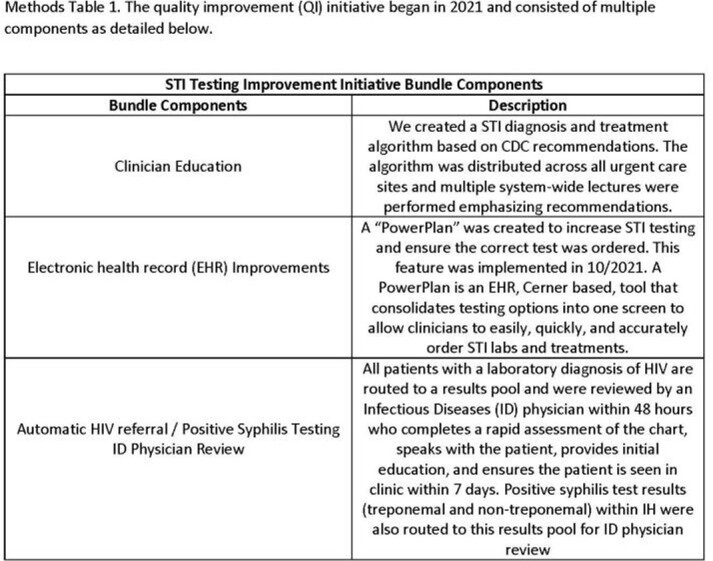
Methods Table 1

The quality improvement (QI) initiative began in 2021 and consisted of multiple components as detailed below.

**Results:**

13,715 and 5,628 UC encounters were associated with GC/CT testing during the pre-intervention and intervention periods, respectively. HIV co-testing was performed in 2,784 (20.3%) GC/CT testing encounters in the pre-intervention period and in 1,674 (29.7%) encounters during the intervention, a relative increase of 37.6%. HIV/syphilis co-testing was performed in 2,304 (16.8%) GC/CT testing encounters and 1,225 (21.8%) encounters during the pre-intervention and intervention phases, respectively. From January 1 2022 – April 1 2022 3 new outpatient HIV diagnoses were identified. The average time from diagnosis to contact with an ID provider was 30.0 hours.

Results Image 1

**Figure:**
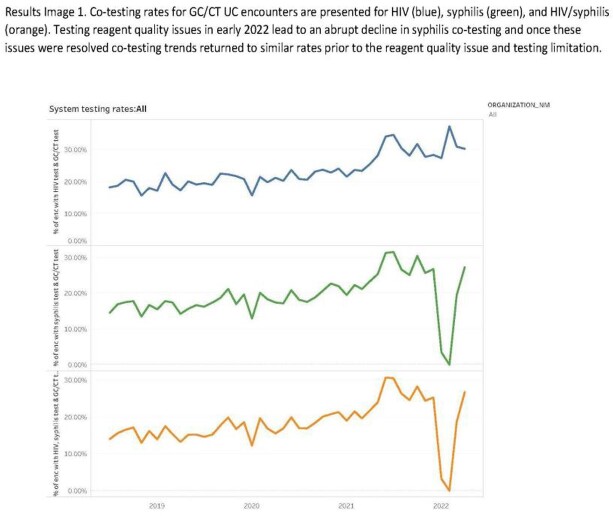

Co-testing rates for GC/CT UC encounters are presented for HIV (blue), syphilis (green), and HIV/syphilis (orange). Testing reagent quality issues in early 2022 lead to an abrupt decline in syphilis co-testing and once these issues were resolved co-testing trends returned to similar rates prior to the reagent quality issue and testing limitation.

**Conclusion:**

Multi-modal QI initiatives may increase STI testing rates within UC centers of integrated healthcare systems. Further study is needed to optimize STI screening, diagnosis, and care in UC centers.

**Disclosures:**

**Kimberly D. Brunisholz, PhD, MST**, Johnson and Johnson: Advisor/Consultant.

